# Effects of Supervised Physiotherapy-Based Exercise on Ovarian Reserve and Spontaneous Pregnancy in Women with Diminished Ovarian Reserve: A Controlled Pilot Study

**DOI:** 10.3390/life16010120

**Published:** 2026-01-13

**Authors:** Barbara Petra Kovács, Júlia Balog, Judit F. Szigeti, Barbara Sebők, Marianna Török, Szabolcs Várbíró

**Affiliations:** 1 Doctoral College, Semmelweis University, 1085 Budapest, Hungary; 2 Workgroup for Science Management, Doctoral College, Semmelweis University, 1085 Budapest, Hungarytorok.marianna@semmelweis.hu (M.T.); varbiro.szabolcs@semmelweis.hu (S.V.); 3 Department of Metabolism, Digestion and Reproduction, Faculty of Medicine, Imperial College London, London W12 0NN, UK; j.balog@imperial.ac.uk; 4 Assisted Reproduction Centre, Department of Obstetrics and Gynecology, Semmelweis University, 1082 Budapest, Hungary; szigeti.f.judit@semmelweis.hu; 5 Department of Otorhinolaryngology, Head and Neck Surgery, Semmelweis University, 1082 Budapest, Hungary; 6 Manninger Jenő Trauma Center, 1081 Budapest, Hungary; 7 Department of Obstetrics and Gynecology, Faculty of Medicine, Semmelweis University, 1082 Budapest, Hungary; 8 Department of Obstetrics and Gynecology, Faculty of Medicine, Szeged University, 6720 Szeged, Hungary

**Keywords:** diminished ovarian reserve, female infertility, physiotherapy-based exercise for improved fertility, spontaneous pregnancy with diminished ovarian reserve, antioxidant supplementation

## Abstract

Diminished ovarian reserve (DOR) is a major cause of female infertility with limited treatment options, and lifestyle interventions such as supervised, structured exercise therapy may support ovarian function. In this pilot study, we evaluated the effect of a supervised, physiotherapy-based exercise program combined with antioxidant supplementation on ovarian reserve markers and spontaneous pregnancy rates in 24 infertile women aged 20–42 years, with body mass index (BMI) 18.5–30 kg/m^2^, regular menstruation, anti-Müllerian hormone (AMH) < 1.1 ng/mL, and antral follicle count ≥3 measured on days 2–4 of the cycle. Participants were randomized into two groups of 12: Both groups received standardized oral therapy, while the intervention group additionally participated in a three-month supervised, structured exercise therapy programme. Analysis of covariance was used to adjust for baseline differences in AMH and BMI, as groups differed significantly in BMI at baseline. At post-treatment assessment, AMH levels were significantly higher in the intervention group, whereas FSH, LH, estradiol, prolactin, and TSH levels did not change significantly. Spontaneous pregnancies were recorded both during the intervention period and throughout a follow-up period of up to six months. Spontaneous pregnancy occurred in 7 out of 12 participants in the intervention group versus 1 out of 12 in the control group, resulting in four and one live births, respectively. These findings suggest that combining supervised, structured exercise therapy with antioxidant supplementation may enhance ovarian reserve and improve the likelihood of spontaneous pregnancy in women with diminished ovarian reserve.

## 1. Introduction

The World Health Organization (WHO) classifies infertility as a disease of the reproductive system, defined by the inability to achieve a clinical pregnancy after at least 12 months of regular, unprotected sexual intercourse. Recent WHO data further indicate that the lifetime prevalence of infertility is approximately 17.5%, while the period prevalence of 12-month infertility is 12.6% [1]. Female infertility is an increasingly pressing global clinical and public health concern [2], with a multifactorial background including age-related ovarian decline [1,3], lifestyle factors such as physical activity patterns [4], and various underlying pathological conditions. Additionally, there is a rising trend toward delayed childbearing [5,6]. Advanced maternal age (AMA)—typically defined as 35 or older—has become increasingly common, contributing significantly to the global rise in subfertility and infertility [7,8], and is associated with a marked decline in natural fertility and poorer assisted reproductive technology (ART) outcomes [9].

Hungarian demographic trends also reflect global patterns of delayed childbearing. In Hungary, the proportion of live births in women over 40 rose from 0.5% in 1990 to 3.3% in 2020 [10]. According to the statistical office of the European Union (EUROSTAT) data, Europe is experiencing a rise in the age at first childbirth, which is associated with a decline in overall fertility rates [5]. A similar trend is observed in the United States [6] and likewise in China [11,12]. These trends underscore the rising demographic weight of postponed parenthood, shaped by social factors and accompanied by reproductive health implications [13].

Diminished ovarian reserve (DOR) is defined by a reduction in both the quantity and quality of oocytes, typically indicated by decreased anti-Müllerian hormone (AMH) levels (<1.1 ng/mL), poor ovarian response to stimulation, and lower pregnancy rates—even during in vitro fertilization (IVF)/intracytoplasmic sperm injection (ICSI) cycles [14,15]. The age-related decline in ovarian reserve can be reliably evaluated through the measurement of anti-Müllerian hormone (AMH), antral follicle count (AFC), and basal follicle-stimulating hormone (FSH) levels [16]. Ovarian reproductive potential can be predicted by both ultrasound imaging of the ovary and biochemical measurements of serum levels of AMH and FSH [17,18].

International data demonstrate that, despite the development of assisted reproductive procedures, live birth rate remains low in the 35 and over age group [19,20,21]. This condition limits reproductive potential and complicates conventional treatment strategies, highlighting the importance of early detection and integrated therapeutic approaches.

The biological aging of the ovaries is a complex and multifactorial process influenced by genetic, environmental, and lifestyle-related factors [22]. In this context, the concept of ovarian rejuvenation—defined as the restoration or significant improvement of previously diminished ovarian function—has garnered increasing attention in both reproductive medicine, particularly through antioxidant-based interventions [23,24] and in regenerative medicine, primarily focusing on intraovarian platelet-rich plasma (PRP) injections and stem cell-based therapies [25,26]. This approach aims to reactivate follicular activity, normalize hormonal profiles, improve ovulation, and, in some cases, lead to spontaneous conception. Despite the growing interest in experimental treatments, no standardized or widely accepted therapy currently exists for age-related diminished ovarian reserve (DOR).

Growing evidence indicates that oxidative stress plays a central role in ovarian aging [27,28], and that antioxidants, as well as moderate-intensity physical activity, can mitigate its effects [29].

It has been shown that supplements have a high impact on the restoration of fertility. Myo-inositol (MI) supplementation has been shown to delay ovarian aging and improve granulosa and cumulus-corona cell function, enhancing oocyte quality by supporting gap junction communication [30,31]. Pre-conceptional folic acid supplementation has been associated with improved female fertility, including higher fecundability compared to women not taking supplements [32]. Melatonin not only regulates sleep but also acts as a potent antioxidant, protecting oocytes from oxidative damage and improving their quality [23,24,33]. Oral intake of 3 mg melatonin daily during assisted reproduction has been shown to increase follicular melatonin levels and reduce oxidative stress [34]. This intervention improves fertilization and pregnancy rates, as well as progesterone production in women with luteal phase deficiency, supporting oocyte quality and luteal function [35]. A combination of melatonin, inositol, and folic acid significantly improved oocyte quality and yield compared to folic acid alone [36]. Higher antioxidant intake, including vitamin C, is positively associated with ovarian reserve in women undergoing fertility treatment [37]. Administration of 200 µg of selenium and 400 IU of vitamin E can reduce ROS overexpression and increase ovarian reserve in patients with primary ovarian insufficiency [38]. Vitamin D_3_ plays a critical role in modulating the immune system and regulating reproductive processes, with deficiencies associated with adverse fertility outcomes [39]. Vitamin D_3_ supplementation may be considered in the fertility treatment of patients with DOR, as it has been demonstrated to cause an increase in AFC and AMH levels and a decrease in FSH levels [40]. Administration of oral coenzyme Q10 prior to ovarian stimulation has been shown to improve IVF/ICSI outcomes in women with diminished ovarian reserve [41].

Sedentary behavior refers to any activity awake characterized by low energy expenditure (≤1.5 metabolic equivalents) in a sitting or reclining posture [42]), whereas physical inactivity is most commonly used to categorize individuals who do not achieve the minimum recommendations of moderate-to-vigorous intensity physical activity (MVPA) (150–300 min/week) [43]. These two behaviors coexist in some cases, and sometimes not [44].

Since the COVID-19 pandemic, global sedentary behavior and physical inactivity have increased [45,46,47], with evidence indicating that women have been affected to a greater extent [48]. This shift in lifestyle patterns raises growing concern about potential impacts on fertility. Sedentary behavior and physical inactivity are two distinct and independent risk factors that should be considered separately in research and clinical practice. According to Foucault’s case–control study, women who spent more than 7 h per day in sedentary behavior had a significantly higher risk of idiopathic infertility [49].

A sedentary and physically inactive lifestyle has detrimental effects not only on reproductive health but also on multiple aspects of mental and physical function. Prolonged sitting negatively impacts postural alignment [50], impairs the spinal stabilizing system [51] and alters respiratory mechanics [52,53]. Extended sitting has been associated with pelvic floor dysfunction and low back pain, primarily due to postural imbalance, reduced core muscle activation, and altered pelvic floor loading [54,55,56,57]. While infertility itself is a significant source of stress, anxiety, and depression [58,59], studies have shown that sedentary behavior is associated with poorer mental health outcomes, including increased symptoms of anxiety and depression [60], and can further exacerbate the psychological well-being of women facing fertility challenges.

Data on the relationship between physical activity and female fertility remain controversial. Although several studies have examined the therapeutic effects of exercise on fertility, no clear consensus has been reached, likely due to the multifactorial nature of fertility [61]. There is also no consensus among researchers on the effect of physical activity before ART on pregnancy outcomes [62,63,64,65].

Moderate-intensity physical activity (PA) has been shown to support ovarian reserve and improve related markers [66], while frequent high-intensity exercise may increase the risk of subfertility, particularly ovulatory infertility [67]. However, some evidence suggests that high-intensity PA may exert a protective effect in women with low baseline activity levels or higher-than-normal BMI [68,69].

The recommendation of the WHO and the American College of Obstetricians and Gynecologists (ACOG) is that women planning a pregnancy should do at least 150 min of moderate physical activity or 75 min of vigorous physical activity, or a mix of the two per week, to reduce reproductive risk [43,70].

In a recently published protocol article [71], we introduced a novel, physiotherapy-based ovarian rejuvenation program designed as a pre-conceptional intervention for women with DOR to synergize with antioxidant and fertility-enhancing supplementation. The protocol outlines a three-arm randomized, open-label trial: Group A receives standard per os antioxidant therapy; Group B, per os therapy plus walking; and Group C, per os therapy combined with a structured, moderate-intensity, physiotherapist-supervised exercise program. The present pilot study represents a partial implementation of this protocol, focusing on Groups A and C. It was conducted with a reduced sample size as a preliminary proof-of-concept study to assess feasibility, program adherence, and potential effects on ovarian reserve markers and spontaneous pregnancy before conducting a larger trial. Our aim is to evaluate the efficacy and safety of the combined intervention and its impact on both spontaneous and ART-related pregnancy outcomes, thereby informing the design of future larger-scale studies.

## 2. Materials and Methods

### 2.1. Study Design

A prospective, two-arm, randomized pilot study was conducted between 2021 and 2025 at Semmelweis University, Budapest, Hungary, to evaluate the effect of a physiotherapy-based exercise program, in combination with oral rejuvenation therapy, on ovarian reserve markers and spontaneous pregnancy in infertile women with DOR.

### 2.2. Recruitment Process

Patients were recruited from the Assisted Reproduction Center of Semmelweis University, Hungary. Detailed information about the study was provided verbally and in writing by the examining physician. We also informed patients that participation in the research is voluntary and that the anonymised data obtained would be summarized and subjected to statistical analysis.

### 2.3. Participants and Eligibility Criteria

All participants were infertile women diagnosed with diminished ovarian reserve according to predefined inclusion criteria. Participants were classified as having primary or secondary infertility based on their reproductive history. In the intervention group, primary infertility predominated (*n* = 9 vs. *n* = 3), whereas primary and secondary infertility were equally distributed in the control group (6:6).

#### 2.3.1. Inclusion Criteria

Participants who met the following inclusion criteria were included: (1) female of reproductive age 20–42 years; (2) BMI: 18.5–30 kg/m^2^; (3) regular menstruation; (4) anti-Müllerian hormone (AMH) < 1.1 ng/mL; (5) understanding the study design, risks and benefits, providing informed consent and (6) ability to comply with the study protocol.

#### 2.3.2. Exclusion Criteria

In a next step, participants meeting any of the following criteria were excluded from the pool: (1) antral follicle count (AFC) < 3 measured on day 2 of the cycle; (2) multiple unsuccessful stimulation cycles ending with cancelation; (3) allergy to medications used for ovarian rejuvenation; (4) three or more ovarian surgeries result in significant ovarian reserve depletion (iatrogenic POI, iatrogenic DOR); (5) uterine developmental abnormalities.

### 2.4. Implementation

The principal investigator or a designated sub-investigator introduces the required data into the web-based allocation system for eligible patients. The required data included: personal data, demographic, medical and family history, anthropometric data (weight, height, BMI), menstrual cycle and hormone levels (AMH, FSH, LH, E2, PRL, TSH, vitamin 25-OH-D3).

### 2.5. Randomization

**Figure 1:**
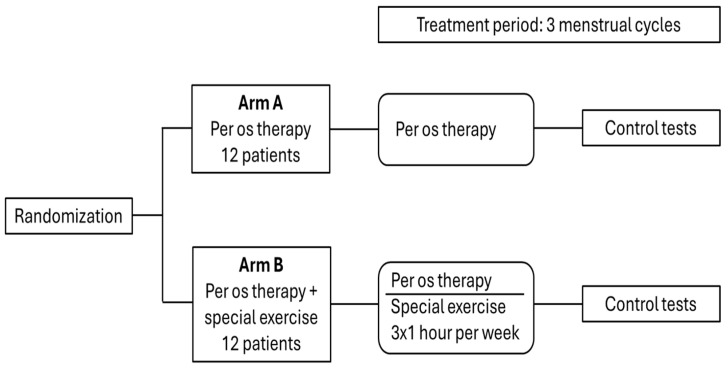
Study design.

Patients were randomly assigned to arm A (per os antioxidant therapy) and arm B (per os therapy and special exercise) in a 1:1 ratio using a computer-based randomization system, with the help of a third-party statistician blinded to the study and participant details. The trial design is summarized in Figure 1.

Blinding of outcome assessment: Laboratory personnel measuring hormone levels were fully blinded to participants’ group allocation, as samples were processed centrally without any information about study participation. AFC measurements were conducted by sonographers who, whenever feasible, were not informed of group assignment. These procedures ensured that outcome assessment was blinded where possible, enhancing the methodological rigor of the study.

### 2.6. Intervention

The standard dietary supplement/vitamin oral therapy for all participant groups included MI + folic acid twice a day, 3 mg of melatonin before bedtime, in addition to daily intake of 1000 mg of vitamin C (slow release), 400 mg of vitamin E, 2500 IU/day of vitamin D3, and 200 mg of CoQ10.

Participants in the control group (Arm A) were instructed to continue their usual daily activities without participating in a structured exercise program. This encompassed routine movements such as commuting, household tasks, and occasional informal physical activity. As this was a pilot study, detailed quantification of habitual activity levels (e.g., via trackers or questionnaires) was not performed, with the primary focus on assessing feasibility and preliminary effects of the supervised intervention.

For Arm B (per os therapy and special exercise), our special 70 min moderate-intensity, physiotherapy-based exercise program was available 3 times a week for at least 3 months. The design of our exercise program aligns with current WHO and ACOG guidelines regarding physical activity for reproductive health. The therapeutic movement program is based on physiotherapeutic principles, integrating elements of yoga, dance therapy, and guided relaxation techniques. This fertility-enhancing exercise program is designed to combine several professional aspects in all exercises including postural correction, pelvic-lumbar stabilization, rehabilitation of the pelvic region, re-education of the pelvic floor muscles, restoration of optimal diaphragm function, dynamic mobilization and stretching exercises, use of the relaxation and therapeutic effects of diaphragmatic breathing (DB), optimization of joint range of motion, development of balance and coordination. Each session is conducted with correct posture and emphasizes not only strengthening key muscle groups but also relaxation and proper stretching of the pelvic and postural muscles to restore neuromuscular balance. Diaphragmatic breathing is mainly used as a relaxation tool. Some exercises also combine DB with pelvic floor muscle (PFM) activation, timed to match the breathing cycle, to improve coordination between these muscle groups and support better functional outcomes. Other exercises are performed using normal breathing. The constant attention on breathing helps the patient in relaxation, activates the parasympathetic nervous system, and ensures the strengthening of multiple muscle groups through contraction and relaxation. A more detailed description of the protocol can be found in [71].

All of these professional aspects, known from yoga and physiotherapy, are complexly reflected in the reproductive exercise program under study. It incorporates quieting the mind through a focus on breathing, precise postures, gentle stretching, diaphragmatic breathing exercises, and body rehabilitation. The uniqueness and complexity of this exercise programme lie in the fact that it integrates both physical and psychological dimensions for effective stress reduction. The program was supervised by a licensed physiotherapist. For the rejuvenation exercise, there was no mandatory equipment; an exercise mat and a pillow/yoga block were recommended.

### 2.7. Outcome Measures

#### 2.7.1. Primary Outcomes

The primary outcomes were: (1) changes in FSH levels compared to baseline; (2) changes in AMH levels compared to baseline; and (3) occurrence of spontaneous pregnancy during the intervention and the 6-month post-intervention follow-up period.

#### 2.7.2. Secondary Outcomes

The secondary outcomes included: post-intervention E2, LH, prolactin, and TSH levels.

### 2.8. Baseline and Follow-Up Assessments

Baseline assessments adhered to the trial’s standard operating procedure (SOP). At the first visit conducted between cycle days 2 and 4, laboratory examinations included measurement of hormone levels: AMH, FSH, LH, E2, PRL, TSH, and 25-OH vitamin D3. All serum reproductive hormone measurements were performed in the same accredited central laboratory using standardized, routinely applied chemiluminescent immunoassay (CLIA) methods in accordance with institutional clinical practice as follows: AMH (Access AMH Advanced kit C62997, Beckmann Access Analyzer, Beckmann Coulter, Brea, CA, USA), TSH, FSH, LH, PRL and E2 (Atellica IM TSH3-UL kit 10995704, FSH kit 10995580, LH kit 10995635, PRL kit 10995656, eE2 kit 10995561, Atellica IM Analyzer, Siemens Healthcare GmbH, Erlangen, Germany), 25-OH D3 (Liaison 25-OH-D-Vitamin Assay 310600, Liason XL Analyzer, DiaSorin Inc., Saluggia, Italy). See Table 1 for a summary.

Throughout the study period, spontaneous pregnancies were monitored. After completion of three consecutive menstrual cycles (post-treatment), hormone levels of AMH, FSH, LH, E2, PRL, and TSH were re-assessed. Spontaneous pregnancies and successful IVF outcomes were followed for an additional 6 months or until live birth (Table 2).

### 2.9. Efficacy Assessments

The efficacy of the trial treatment was evaluated using a comprehensive set of parameters measured at the 3–6-month follow-up. Participants were followed for a total of approximately 9 months, including an intervention period corresponding to three consecutive menstrual cycles, followed by an additional 6 months of follow-up or until a live birth if a spontaneous pregnancy occurred. Spontaneous pregnancy was self-reported and confirmed by serum β-hCG testing and/or transvaginal ultrasound at 6–8 weeks of gestation. No assisted reproductive technologies were used during the study period.

### 2.10. Statistical Analysis

All statistical analyses were performed using GraphPad Prism (version 10.3.1.509, GraphPad Software, San Diego, CA, USA) and IBM SPSS Statistics (version 25, IBM Corp., Armonk, NY, USA). Continuous variables were tested for normality using the Shapiro–Wilk test. Variables with normal distribution are presented as mean ± standard error of the mean (SEM), while non-normally distributed variables are presented as median with 95% confidence interval (CI). Categorical variables are reported as absolute numbers and percentages.

Baseline characteristics were compared between groups using independent *t*-tests or Mann–Whitney U-tests for continuous variables and Fisher’s exact tests for categorical variables. Within-group pre–post changes were evaluated using paired *t*-tests or Wilcoxon signed-rank tests, as appropriate.

For all hormonal outcomes (AMH, FSH, LH, E2, PRL, and TSH), analysis of covariance (ANCOVA) was performed using SPSS to compare follow-up values between the treatment (exercise + oral therapy) and control (oral therapy only) groups, while adjusting for baseline values and body mass index (BMI). In each model, the follow-up measurement was the dependent variable, the study group was the fixed factor, and the baseline value and BMI were entered as covariates. This adjustment allowed estimation of between-group differences that were independent of initial hormone levels and BMI. Adjusted mean differences and their 95% confidence intervals (CI) are reported.

All statistical tests were two-sided, and a *p*-value < 0.05 was considered statistically significant. Given the pilot nature of the study, no formal sample size calculation was performed. The primary aim was to assess feasibility, compliance, and to generate preliminary effect size estimates to inform the design of a future larger randomized controlled trial.

### 2.11. Ethics Approval

The study protocol was approved and registered by the Human Reproduction Committee of the Hungarian Medical Research Council (25489-8/2021/EÜIG), dated 12 July 2021, and was conducted in accordance with the Declaration of Helsinki. All participants provided written informed consent prior to participation.

## 3. Results

Twenty-four women aged 20–42 years with infertility and diminished ovarian reserve were enrolled in the treatment (*n* = 12) and the control (*n* = 12) group. No discontinuation of the trial was registered in either group.

A comparison of baseline data in the control and treatment groups (Table 3) revealed no significant differences in age or initial hormone levels between groups; however, BMI was significantly higher in the control group than in the treatment group. The post-treatment levels at 3 months show significant improvement in AMH levels in the treatment group compared to the control group, with no other significant changes in hormone levels.

**Figure 2:**
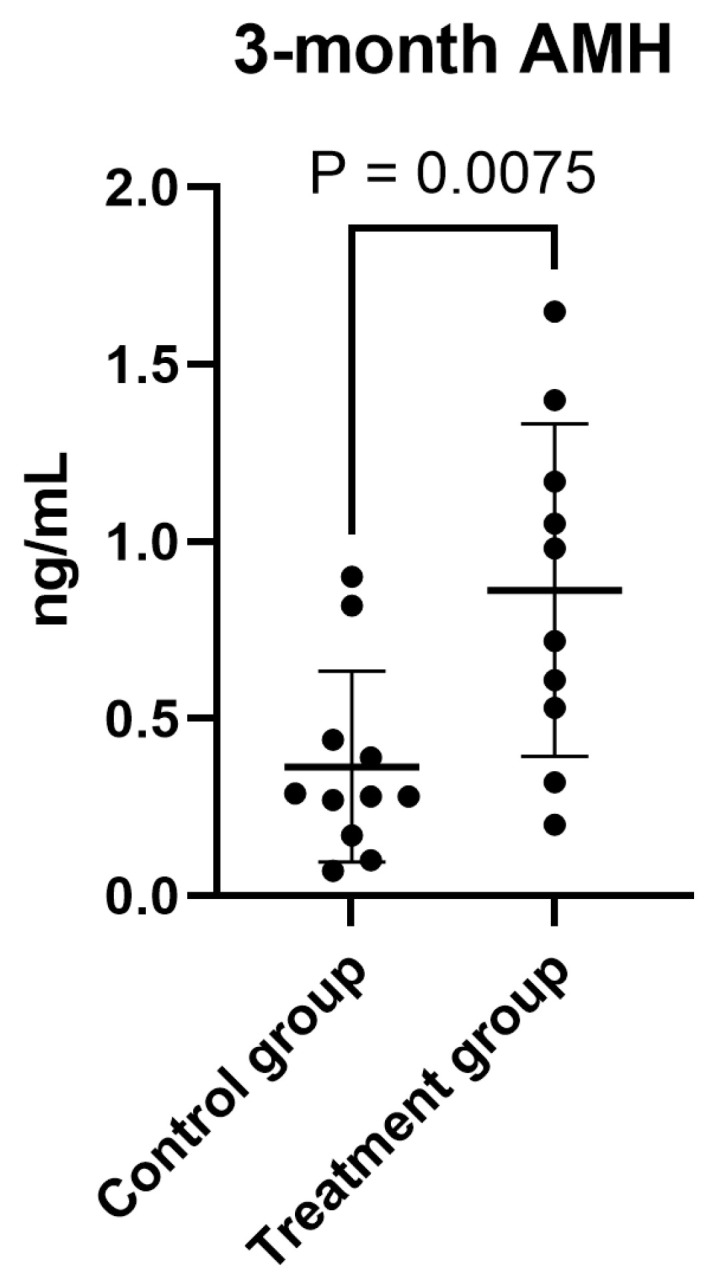
Serum AMH levels post-treatment in the treatment (exercise + oral therapy) and control (oral therapy only) groups. Data are presented as median (95% CI). The between-group difference in unadjusted analysis (Mann–Whitney test) was statistically significant (*p* = 0.0075). In a multivariate ANCOVA model including all hormonal outcomes, spontaneous pregnancy, and age, and adjusting for baseline AMH and BMI, the difference remained significant (*p* = 0.008). Sample size: *n* = 12 per group.

**Figure 3:**
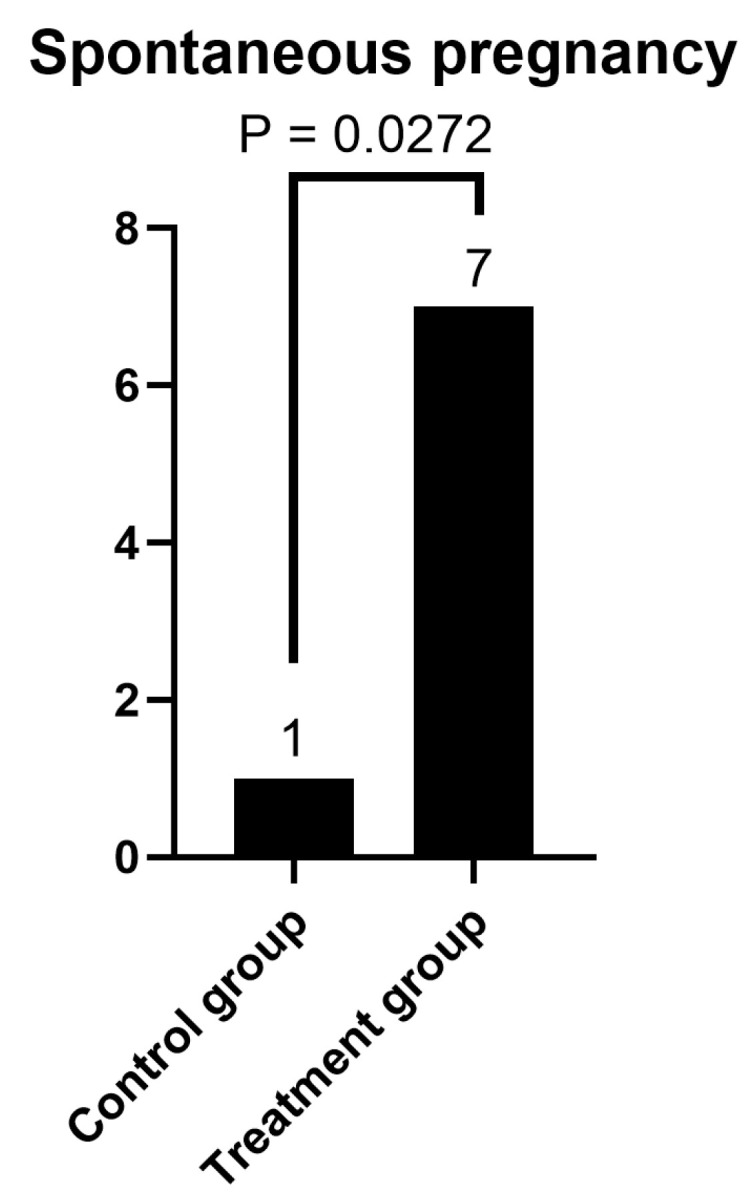
Spontaneous pregnancy rates during the intervention and follow-up periods in the treatment (exercise + oral therapy) and control (oral therapy only) groups. Bars indicate the number of women with and without spontaneous pregnancy in each group. The between-group difference in unadjusted analysis (Fisher’s exact test) was statistically significant (*p* = 0.0272). In a multivariate ANCOVA model including all hormonal outcomes, spontaneous pregnancy, and age, and adjusting for baseline AMH and BMI, the difference remained significant (*p* = 0.013). In a separate ANCOVA model including only spontaneous pregnancy as the outcome and adjusting for BMI and baseline AMH, the difference also remained significant (*p* = 0.034), and BMI had no effect (*p* = 0.998). Sample size: *n* = 12 per group.

To avoid BMI-induced distortion, we performed an ANCOVA analysis. When examining hormones, pregnancy, and age, taking into account BMI and initial AMH levels, AMH was significantly higher in the treatment group than in the control group at the post-intervention assessment (Figure 2). The incidence of spontaneous pregnancy during the intervention and follow-up periods was significantly higher in the treatment group than in the control group (Fisher’s exact test, *p* = 0.0272; Figure 3). In the multivariate ANCOVA model described above, the difference remained significant (*p* = 0.013).

Spontaneous pregnancies were observed in 7 of 12 participants in the treatment group and in 1 of 12 participants in the control group. In the treatment group, three spontaneous pregnancies occurred during the intervention period, none of which resulted in a live birth. Four additional spontaneous pregnancies occurred during the 6-month post-intervention follow-up period, all resulting in live births. In the control group, one spontaneous pregnancy occurred during the study period and resulted in a live birth. Overall, four live births were recorded in the treatment group and one in the control group.

No significant between-group differences were observed after 3 months in FSH, LH, E2, prolactin, or TSH levels in either the unadjusted or ANCOVA analyses (Table 2).

We performed an ANCOVA test only on AMH levels, taking BMI into account, and again found that AMH was significantly higher in the treated group than in the control group at the post-treatment assessment. While the data suggests a potential increase in AMH levels following exercise, the limited sample size precludes definitive conclusions, and the observed difference may be attributable to random variation.

The ANCOVA was also performed separately for spontaneous pregnancies, with BMI and baseline AMH included as covariates. The difference in spontaneous pregnancy rates between the treatment and control groups and the effect of BMI were also filtered out here. When spontaneous pregnancy was analyzed separately in an ANCOVA model adjusting for BMI and baseline AMH, the between-group difference was still significant (*p* = 0.034), whereas BMI was completely irrelevant (*p* = 0.998). In other words, exercise did indeed increase the likelihood of a spontaneous pregnancy, and this was not caused by differences in BMI.

In summary, participation in the exercise program over three consecutive menstrual cycles, in addition to oral rejuvenation therapy, was associated with higher AMH levels and an increased likelihood of spontaneous pregnancy, even after adjusting for baseline AMH and BMI. Although the exercise program did not significantly affect other hormonal parameters, a declining tendency was observed in the exercise group.

The intervention was well-tolerated and feasible, with participants attending over 85–90% of the scheduled exercise sessions. Missed sessions were primarily due to minor reasons such as 1–2 days of painful menstruation, a few days of febrile illness, or short vacations (up to one week). These data demonstrate a high level of engagement and support the practicality of implementing this supervised program in future larger trials.

## 4. Discussion

To date, most interventional studies on exercise and female fertility have focused on women with polycystic ovary syndrome. In this population, structured exercise programs—including moderate-intensity continuous training and high-intensity interval training (HIIT)—have improved insulin sensitivity, menstrual cyclicity, androgen levels, and in some cases, ovulation and pregnancy outcomes [72,73]. Lifestyle interventions combining exercise with dietary or pharmacological strategies show the strongest effects on BMI reduction, ovulatory function, and hormonal normalization [74], while also enhancing psychological well-being and quality of life [75]. In contrast, women with diminished ovarian reserve (DOR) are largely understudied. Although exercise physiology and reproductive endocrinology suggest that physical activity can influence hypothalamic-pituitary-ovarian function [76], there is a lack of interventional studies evaluating physiotherapy-based exercise in this patient group.

The role of BMI in reproductive outcomes remains controversial, as several studies have found no significant effect on pregnancy rates. A large cohort study including more than 30,000 women likewise reported no association between BMI and conception rates, further supporting the limited relevance of this parameter in this context. Therefore, in interpreting our results, we note that the available evidence does not support a major impact of BMI [77,78]. Regardless of the controversial studies and statistical adjustment for BMI, this pilot study results should be interpreted with caution. The intervention (mean BMI 25.6, overweight range) and control (mean BMI 21.5, normal range) groups differ biologically in BMI, which may influence reproductive outcomes independently of statistical correction. This limitation should be considered when extrapolating findings and planning future larger studies

Although no statistically significant difference in age was observed between the groups, the higher mean age in the control group (38.0 vs. 35.3 years) falls within an age range associated with a progressive decline in female fertility. Therefore, a potential biological influence of age on the observed outcomes cannot be entirely excluded. According to a large-scale meta-analysis, the probability of natural conception after 6 months declines from 15% to 10% between the ages of 35 to 38 [79].This could possibly account for one extra spontaneous pregnancy in the treatment group; however, this does not change the significant difference between the two groups.

Although numerous studies have explored the relationship between physical activity and fertility, direct comparisons remain challenging due to considerable heterogeneity in program structure, target populations, inclusion and exclusion criteria, and the duration and nature of the interventions and assessment methods. Our literature review did not identify any physiotherapy-based interventions of comparable complexity, making it difficult to directly compare our findings with previous results.

Our pilot study, therefore, fills a critical knowledge gap, demonstrating feasibility and providing preliminary effect-size estimates to guide future adequately powered randomized trials assessing the impact of physiotherapy-based exercise on ovarian function and fertility in women with DOR.

In our two-arm pilot study (oral therapy vs. oral therapy plus supervised physiotherapy-based exercise), AMH levels were significantly higher in the intervention group post-treatment, remaining significant after BMI-adjusted ANCOVA. Spontaneous pregnancy incidence over six months was also higher in the intervention group, and this difference persisted after adjustment for BMI in multivariable ANCOVA. No between-group differences were observed for FSH, LH, E2, prolactin, or TSH at three months. These findings suggest that a supervised physiotherapy-based program may support ovarian function and natural fertility in women with DOR.

Potential mechanisms underlying these effects may include enhanced pelvic blood flow, hormonal regulation, lumbopelvic stability, and stress reduction. Yoga-based interventions have been shown to improve uterine artery perfusion [80], while changes in vascular resistance observed in hypoestrogenic amenorrhea further emphasize the role of circulation [81]. Exercise has also been associated with favorable alterations in ovarian reserve markers and fertility outcomes [66,68,82].

Coordinated activation of the diaphragm, pelvic floor, transversus abdominis (TVA), and multifidus is essential for lumbopelvic stability, posture, and the optimal positioning of reproductive organs [83]. The pelvic floor regulates intra-abdominal pressure in synergy with core muscles [52], and targeted strengthening significantly improves pelvic floor function, organ support [84]. Activation peaks in the lumbar neutral position [85], highlighting the importance of posture. Respiration-related activation of pelvic floor muscles contributes to postural control [86], while diaphragmatic breathing combined with trunk stability exercises effectively restores stability after core fatigue [87]. Rehabilitation protocols integrating lumbopelvic stabilization and diaphragmatic breathing may therefore support reproductive health by addressing both mechanical and neurophysiological components.

Women undergoing fertility treatment frequently experience infertility-specific stress, reflecting the emotional burden of reproductive challenges rather than causing infertility directly [88]. Diaphragmatic breathing (DB) and yoga-based mind–body interventions are effective for stress reduction: DB activates the parasympathetic system [89], while yoga reinforces resilience and may improve reproductive outcomes [90,91]. Proper DB not only reduces stress but also supports trunk and pelvic stabilization [87], potentially enhancing the efficacy of targeted exercise interventions on ovarian reserve and fertility. Additionally, moderate-intensity exercise may improve antioxidant defenses and restore redox balance, further supporting ovarian function [29,92].

Overall, physiotherapy-based interventions may enhance reproductive health via improved circulation, lumbar-pelvic stability, stress reduction, and oxidative balance, offering a safe, non-invasive complement to conventional fertility care. This investigation provides a unique perspective by bridging conventional and integrative therapeutic approaches. It aims to establish a non-invasive, evidence-based adjunct to fertility care, contributing to the development of personalized, lifestyle-integrated strategies that support ovarian function and improve reproductive outcomes in women with diminished ovarian reserve.

### 4.1. Strengths

Key strengths of the present study include the controlled, two-arm design; non-invasive nature of the intervention with no necessity of hormonal therapy or strong drug treatment; minimal to no chance of side effects; professionally supervised, physiotherapist-led sessions ensuring correct technique and high adherence; and a comprehensive outcome set spanning primary (AMH, FSH, spontaneous pregnancy) and secondary (LH, E2, TSH, prolactin, IVF outcomes) endpoints, with a six-month follow-up for pregnancies and IVF results. This integrated design enhances internal validity and also helps to better understand the underlying physiological mechanisms behind the observed outcomes.

### 4.2. Limitations

Limitations include the small sample size (*n* = 24) and limited follow-up, which restrict statistical power and the drawing of long-term inference; results should therefore be interpreted as preliminary indications of feasibility and efficacy rather than definitive evidence. A significant difference in mean BMI between the intervention (25.6, overweight range) and control (21.5, normal range) groups represents an important limitation. Although statistical adjustments were applied, this difference may influence reproductive outcomes independently of the exercise intervention, making it difficult to attribute the observed effects solely to the physiotherapy-based exercise program.

Although the distribution of primary and secondary infertility differed between groups, the predominance of primary infertility in the intervention group would be expected to bias outcomes toward poorer prognosis rather than inflate the observed effects.

Accordingly, the reported improvements in ovarian function and spontaneous conception are unlikely to be attributable to this baseline difference.

### 4.3. Clinical and Practical Significance

The intervention is simple, non-invasive, and well-tolerated, and can be integrated with conventional fertility treatments. Given the emotional and physical burden of infertility, a supervised physiotherapy-based program may offer adjunctive biological and psychosocial benefits for women with DOR.

### 4.4. Future Research Directions

Future trials should be adequately powered and of longer duration to be considered. Monitoring additional biomarkers to clarify mechanisms, including markers of oxidative stress (e.g., malondialdehyde), inflammation (e.g., high-sensitivity C-reactive protein [hs-CRP]), and insulin resistance (e.g., HOMA-IR), measured at baseline and post-intervention, would also be beneficial. Imaging and protocol variations (e.g., intensity, frequency, component emphasis) could also be explored. Such studies are needed to establish evidence-based guidance for integrating physiotherapy-based programs into fertility care for DOR.

## 5. Conclusions

This pilot study provides preliminary, hypothesis-generating evidence that a structured, supervised physiotherapy-based exercise program, combined with antioxidant and fertility-enhancing supplementation, may be associated with improvements in ovarian function and spontaneous pregnancy rates in women with diminished ovarian reserve. Observed improvements in AMH suggest potential benefits that may translate into clinically meaningful reproductive outcomes, possibly mediated through improved pelvic circulation, neuromuscular support, and stress reduction. Importantly, the intervention was feasible, well-tolerated, and non-invasive, highlighting its potential applicability as an adjunctive supportive strategy in fertility care. While limited by the small sample size and relatively short follow-up period, these findings underscore the potential clinical relevance of targeted physiotherapy-based interventions in a population with limited therapeutic options. Further investigation in larger, adequately powered trials is necessary to confirm potential efficacy and to explore underlying mechanisms, without implying definitive causality or replacement of standard fertility treatments.

## Figures and Tables

**Table 1:** Serum hormone and vitamin measurements. High (>10%) intra-assay cross variance is due to low sample concentrations.

Assay	Kit/Catalog #	Platform	Sensitivity/Measuring Interval	Intra-Assay CV (%)
Access AMH Advanced	C62997	Beckman Coulter Access/DxI	0.08–24 ng/mL (≈0.57–171 pmol/L)	1.5–10
Atellica IM TSH3-UL	10995704	Siemens Healthineers Atellica IM	0.008–150 μIU/mL	2–16
Atellica IM FSH	10995580	Siemens Healthineers Atellica IM	0.30–200 mIU/mL	2–10
Atellica IM LH	10995635	Siemens Healthineers Atellica IM	0.07–200 mIU/mL	2–10
Atellica IM Prolactin (PRL)	10995656	Siemens Healthineers Atellica IM	0.47–200 ng/mL (latest IFU rev 06, 2024-06)	2–12
Atellica IM Enhanced Estradiol (eE2)	10995561	Siemens Healthineers Atellica IM	11.80–3000 pg/mL	2–20
LIAISON^®^ 25-OH Vitamin D TOTAL	310600	DiaSorin LIAISON	4–150 ng/mL	Serum: 2.9–5.5; Plasma: 3.2–8.1

**Table 2:** Study assessment and procedures.

	Screening	Baseline (Cycle Days 2–4)	Post-Treatment (3 Cycles; Cycle Days 2–4)	Follow-Up (Up to 6 Months)
Investigator meeting	✓			
Informed consent	✓			
Demographics, medical, and family history	✓			
Anthropometric data (weight, height, BMI)	✓			
Menstrual cycle	✓			
Hormone levels (AMH, FSH, LH, E2, PRL, TSH, 25-OH-D3 vitamin)		✓	✓	
AFC		✓		
Spontaneous pregnancy			✓	✓
IVF outcome				✓

Abbreviations: AMH = anti-Müllerian hormone; FSH = follicle-stimulating hormone; LH = luteinizing hormone; E2 = estradiol; PRL = prolactin; TSH = thyroid-stimulating hormone; 25-OH-D3 = 25-hydroxyvitamin D; AFC = antral follicle count; BMI = body mass index; IVF = in vitro fertilization.

**Table 3:** Baseline and post-treatment hormonal parameters in the treatment and control groups. Data are presented as mean ± SEM for normally distributed variables and median (95% CI) for non-normally distributed variables, as determined by the Shapiro–Wilk test. Between-group differences were assessed using independent *t*-tests or Mann–Whitney tests, as appropriate.

	Control Group	Treatment Group	*p*-Value
Age	38.00 ± 0.60	35.25 ± 1.47	0.104
BMI	25.64 ± 0.96	21.49 ± 0.61	0.0014
AMH	0.50 ± 0.07	0.54 ± 0.08	0.695
FSH	10.83 ± 0.74	12.16 ± 1.36	0.397
LH	4.83 ± 0.69	5.82 ± 0.57	0.281
E2	42.25 (38.00–62.20)	40.01 (25.30–63.70)	0.370
prolactin	11.51 (8.45–33.62)	11.29 (7.24–15.30)	0.525
TSH	2.06 ± 0.29	1.83 ± 0.20	0.515
3-month AMH	0.28 (0.10–0.82)	0.85 (0.32–1.40)	0.0075
3-month FSH	13.07 ± 2.04	9.39 ± 0.90	0.122
3-month LH	4.30 (2.82–10.40)	4.55 (3.50–7.00)	0.877
3-month E2	47.60 (34.10–95.70)	43.45 (33.70–80.95)	0.349
3-month prolactin	10.85 ± 1.143	9.90 ± 1.82	0.659
3-month TSH	1.47 (0.21–3.86)	1.48 (1.08–3.54)	>0.999

Abbreviations: AMH = anti-Müllerian hormone; FSH = follicle-stimulating hormone; LH = luteinizing hormone; E2 = estradiol; TSH = thyroid-stimulating hormone; BMI = body mass index. Control group = oral therapy only; Treatment group = oral therapy + supervised physiotherapy-based exercise. Values are mean ± SD or median (range); *p*-values indicate between-group differences.

## Data Availability

The dataset supporting the conclusions of this article is included within the article.

## Abbreviations

The following abbreviations are used in this manuscript:

AFC	antral follicle count
AMH	anti-Müllerian hormone
ANCOVA	analysis of covariance
ART	assisted reproductive technology
BMI	body mass index
CI	confidence interval
DB	diaphragmatic breathing
DOR	diminished ovarian reserve
E2	estradiol
FSH	follicle-stimulating hormone
IVF	in vitro fertilization
LH	luteinizing hormone
PRL	prolactin
TSH	thyroid-stimulating hormone
TVA	transversus abdominis
